# Further investigation of the effects of wearing the hijab: Perception of female facial attractiveness by Emirati Muslim men living in their native Muslim country

**DOI:** 10.1371/journal.pone.0239419

**Published:** 2020-10-21

**Authors:** Timothy R. Jordan, Hajar Aman Key Yekani, Mercedes Sheen

**Affiliations:** 1 Department of Psychology, Ibn Haldun University, Istanbul, Turkey; 2 Department of Psychology, Heriot-Watt University, Dubai, UAE; Macquarie University, AUSTRALIA

## Abstract

The hijab is central to the lives of Muslim women across the world but little is known about the actual effects exerted by this garment on perceptions of the wearer. Indeed, while previous research has suggested that wearing the hijab may affect the physical attractiveness of women, the actual effect of wearing the hijab on perceptions of female facial attractiveness by Muslim men in a Muslim country is largely unknown. Accordingly, this study investigated the effects of the hijab on female facial attractiveness perceived by practising Muslim men living in their native Muslim country (the United Arab Emirates). Participants were presented with frontal-head images of women shown in three conditions: in the *fully covered* condition, heads were completely covered by the hijab except for the face; in the *partially covered* condition, heads were completely covered by the hijab except for the face and areas around the forehead and each side of the face and head; in the *uncovered* condition, heads had no covering at all. The findings revealed that faces where heads were uncovered or partially covered were rated as equally attractive, and both were rated as substantially more attractive than faces where heads were fully covered. Thus, while wearing the hijab can suppress female facial attractiveness to men, these findings suggest that not all hijab wearing has this effect, and female facial attractiveness for practising Muslim men living in their native Muslim country may not be reduced simply by wearing this garment. Indeed, from the findings we report, slight changes to the positioning of the hijab (the *partially covered* condition) produce perceptions of facial attractiveness that are no lower than when no hijab is worn, and this may have important implications for wearing the hijab in Muslim societies. Finally, we argue that the pattern of effects we observed is not explained by anti-Islamic feeling or cultural endogamy, and that a major contributory factor is that being fully covered by the hijab occludes external features, especially the hair and lateral parts of the head and face, which, when normally visible, provide a substantial perceptual contribution to human facial attractiveness.

## Introduction

**Fig 1:**
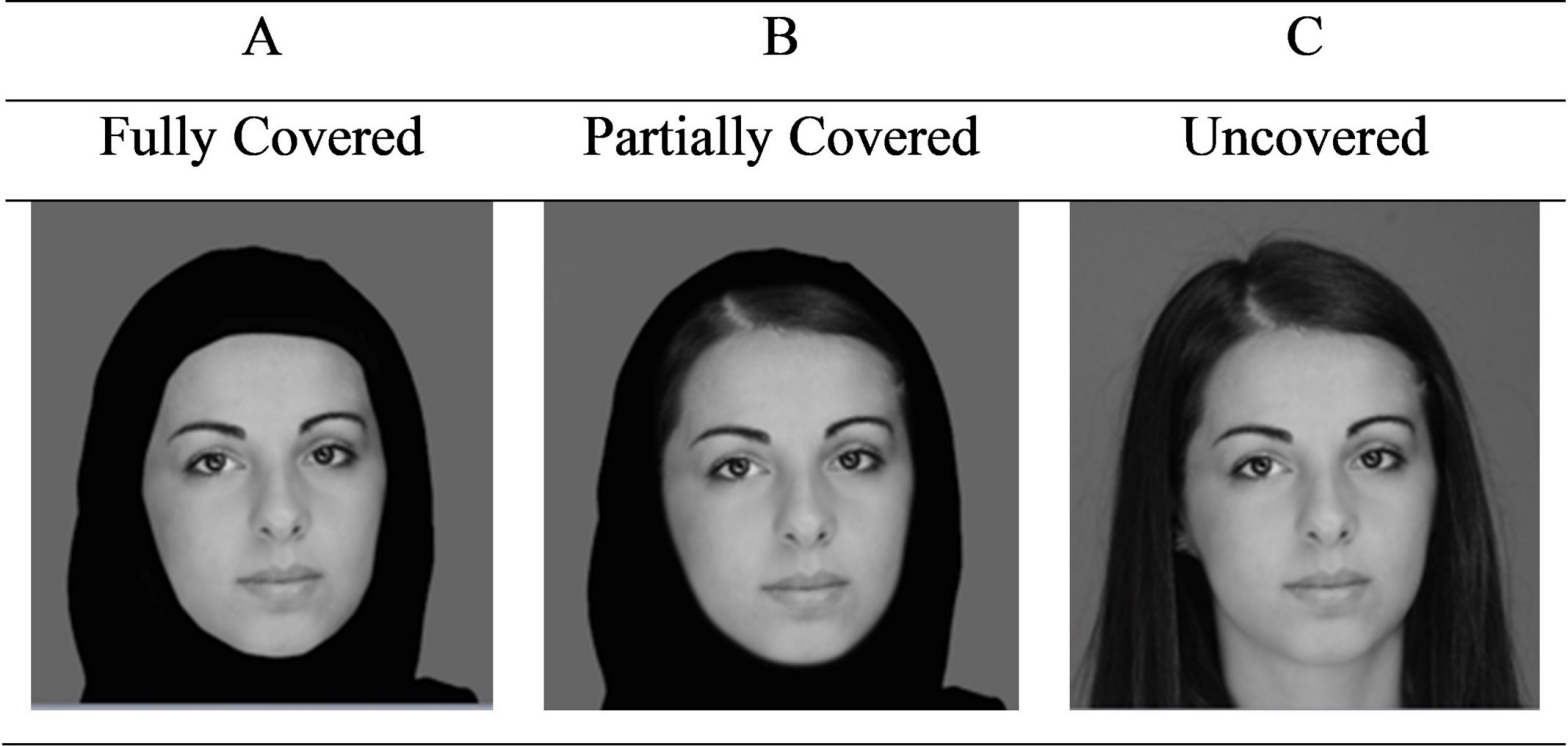
Examples of stimuli used in the three display conditions of this study. N.B. The individual shown gave written informed consent to publish these images. (Image originally published by Sheen, Yekani, & Jordan [6] under CC BY).

The hijab is a traditional head covering worn by Muslim women across the world as a symbol of modesty, piety, and cultural identity. Indeed, for many Muslim women, the hijab acts as a clear expression of their faith and is a major factor in being identified as Muslim. Moreover, although the way in which the hijab is worn can vary (e.g., tightly around the face or more loosely; Fig 1A and 1B), millions of Muslim women, and especially those native to the United Arab Emirates (UAE), choose to wear this head covering when in public (e.g., [1–4]). In fact, many regard the hijab as a means of limiting the attentions of men, and argue that this is reflected in the writings of the *Qur'an* (e.g., verse 24:31; see [5] for interesting discussions).

The personal and social aspects of religious veiling have been studied extensively but the actual function of religious veiling remains a matter of some debate. From an evolutionary perspective, for example, veiling may reduce female attractiveness and act as a mate guarding strategy [7; see also 8, 9] which helps safeguard a man’s access to a female mate while simultaneously preventing advances from rival men. But although the use of religious veiling to reduce female attractiveness may have a clear intentional function, the actual effectiveness of the hijab in influencing how men perceive the attractiveness of the female wearer remains to be fully understood.

A growing body of scholarly work has sought to reveal the influence that the hijab actually produces on perceptions of the wearer. But while some research has been conducted on the effect of the hijab on processes such as recognizing faces [10] and implicit bias [11], few studies [3, 6, 12, 13] have investigated whether wearing the hijab actually has any effect on perceptions of facial attractiveness. Indeed, and perhaps due to this dearth of research, the effects of the hijab on perceptions of the facial attractiveness of women are still far from clear.

In one of these studies (Pasha-Zaidi [3]), two groups of South Asian Muslim women, one group living in the UAE and one in the USA, were shown full face images of White and South Asian women either wearing the hijab or completely uncovered, and participants were asked to rate the attractiveness of each image. Overall, the findings indicated that images of faces where the hijab was worn were rated as *more* attractive by both groups of participants, suggesting that wearing the hijab generally increases facial attractiveness to other Muslim women. But this apparent preference is complicated by other aspects of this study. First, while using participants who originated from South Asia (mostly India, Pakistan, and Bangladesh) would not necessarily be problematic in the USA, social distinctions in the UAE between native, national citizens (Emiratis) and non-native residents are substantial, and South Asian women in the UAE tend to hold low-status positions, have low job security, and experience considerable risk if they contravene UAE laws or customs [14]. In contrast, the majority of Emiratis (males and females) enjoy very high social status in the UAE, and Emirati women habitually wear the hijab in public. Indeed, although the population of the UAE is over 9 million people, Emiratis make up only around 11% of this number [15], and Emiratis (male and female) regard wearing the hijab as a symbol of national pride, cultural identity, and a way of distinguishing themselves as being “local” [16]. Thus, in the UAE particularly, the hijab provides important symbolic status because it signifies not only faithfulness to culture, tradition, and religion, but also access to the benefits and privileges of “belonging” to the UAE [17, 18]. As a result, the higher facial attractiveness ratings for covered facial images reported by Pasha-Zaidi [3] for non-native participants in the UAE may reflect participants’ deference to a higher social status group [19] and a desire to respond positively towards this group [20], rather than genuine views on attractiveness. Indeed, some support for this comes from other aspects of the same study reported by Pasha-Zaidi where, in the USA, only hijab-wearing participants rated images of covered faces as more attractive than uncovered, whereas, in the UAE, participants rated images of covered faces as more attractive regardless of their own hijab-wearing status.

But other concerns about the findings of Pasha-Zaidi [3] are raised by the use of web-based questionnaires to collect data, and the use of a snowballing technique to recruit participants. Although snowball sampling is sometimes regarded as an effective way of reaching out to “hidden” populations [21], it can also result in a biased, unknown, and non-representative sample with little control over who actually completes the surveys and provides the data (see also discussions by [22, 23]). The use of web-based questionnaires generally is also problematic as data gathered in this way can be adversely affected by inattentive or non-serious responses which further undermine the validity of the results (e.g., [24, 25]). So although Pasha-Zaidi’s results are interesting, it is difficult to be certain that the attractiveness ratings collected were made by appropriate participants or accurately reflected the perceived facial attractiveness of women wearing the hijab. Finally, the facial images used by Pasha-Zaidi were not matched when wearing and not wearing the hijab as each face was photographed separately in these two stimulus conditions. In these situations where different photographs are used, perceptible differences in facial expression occur easily and this confound is apparent in the example images provided by the Pasha-Zaidi article. Without matching precisely the facial images used across different stimulus conditions, the effect of wearing the hijab on facial attractiveness cannot be established with acceptable levels of accuracy.

These complications with the study by Pasha-Zaidi [3] were addressed recently by our group (Sheen, Yekani, & Jordan [6]). First, we investigated the effect of the hijab on perception of facial attractiveness by participants who were practicing Muslim women living in their native Muslim country (the UAE) where Islam and wearing the hijab are normal, accepted, and widespread aspects of everyday life, and where non-native or anti-Islamic feelings should not influence participants’ judgments. Second, all participants were native Emiratis whose personal details and background had been screened carefully for inclusion to ensure that their ratings would provide a genuine assessment of how the hijab affects perceptions by native Emiratis in the UAE. In addition, all participants took part under closely controlled experimental conditions. Finally, care was taken to match facial images precisely across covered and uncovered conditions to provide an accurate measure of facial attractiveness in each condition that was not contaminated by differences in facial expression. Examples of the images used in this study are shown in Fig 1. The findings showed that faces in images where heads were fully covered by the hijab were rated as significantly less attractive than faces in images where heads were uncovered. Similar detrimental effects were observed even when heads were only partially covered by the hijab (see Fig 1). These findings suggest that, even for practising Muslim women living in their native Muslim country and for whom wearing the hijab and seeing others wearing the hijab are normal aspects of everyday life, perception of facial attractiveness is lowered when this garment is worn. Thus, while wearing the hijab may be influenced by male attitudes towards suppressing female attractiveness to men ([7–9; see also 26]), the findings from this study suggest that female Muslims too experience the negative influence of wearing the hijab on perception of female facial attractiveness.

However, these studies by Pasha-Zaidi [3] and Sheen et al. [6] were not concerned with the effect of the hijab on facial attractiveness perceived by men, and the remaining two previous studies to have investigated the influence of wearing the hijab on facial attractiveness [12, 13] examined this issue. In both studies, Muslim and non-Muslim British males living in the UK were required to rate the facial attractiveness of images in which women’s heads were displayed either completely uncovered or covered by the hijab so that only the face was visible. Overall, faces where the hijab was worn were rated by Muslims and non-Muslims as less attractive than when women were uncovered, although this effect was greatest for non-Muslims. Moreover, whereas Muslims and non-Muslims showed no significant difference in attractiveness ratings for faces when the hijab was worn, non-Muslims gave higher attractiveness ratings than Muslims for faces when women were uncovered. Unfortunately, as in the study by Pasha-Zaidi [3], these findings by Mahmud and Swami [12] and Swami [13] are complicated by the use of facial images that were not matched when wearing and not wearing the hijab, and perceptible differences are evident in the examples given by Mahmud and Swami [12]. Nevertheless, the authors of both studies make the reasonable suggestion that the patterns of facial attractiveness observed were affected greatly by the country in which the research was conducted (the UK) where negative perceptions of Islamic symbols, such as the hijab, place Muslims as outsiders and encourage feelings of prejudice and acts of discrimination (e.g., [27]; see also [28]). Indeed, it is also suggested that these influences may even have affected perceptions by Muslim men in the UK who unconsciously internalized anti-Islamic messages that have been prevalent in Western media since the 9/11 and 7/7 attacks in New York and London, respectively, and this also affected the attractiveness ratings that were obtained.

But because the hijab is likely to act as a marker for anti-Islamic stigmatization in the UK (and in other countries where Islam is not the dominant culture and religion; for reviews, see [11, 29]), it remains to be determined how wearing the hijab actually affects male perceptions of female facial attractiveness in an environment where this stigmatization is unlikely to be an influence. Of particular importance is that, in contrast to perceptions made by Muslim men in a non-Muslim country [12, 13], a direct measure of the influence of the hijab on perception of female facial attractiveness by Muslim men in the absence of anti-Islamic influences is most likely to be provided by Muslim men living in their native Muslim country, where participants’ judgments should not be influenced by anti-Islamic feelings.

Accordingly, the aim of the present research was to increase our understanding of the effect of wearing the hijab on Muslim men’s perceptions of female facial attractiveness by using the improved methodology and procedures developed by our group [6] to extend previous research in several important ways. First, in contrast to the research conducted with Muslim men in a non-Muslim country (the UK; [12, 13]), we investigated the effect of the hijab on perception of female facial attractiveness by practicing Muslim men in their native Muslim country (the UAE), where Islam and wearing the hijab are normal, accepted, and widespread aspects of everyday life. Second, in contrast to the study by Pasha-Zaidi [3], the personal details and backgrounds of all participants were screened carefully to ensure that all participants lived in and were native to the UAE so that their responses would provide an authentic assessment of how the hijab affects perceptions of facial attractiveness by native Muslim men. In addition, participants took part in the experiment under closely-controlled procedural conditions. Third, facial images were matched precisely across stimuli where the hijab was worn and not worn, in order to provide an accurate assessment of the effect of the hijab on facial attractiveness that was not confounded by differences in facial expression (cf. [3, 12, 13]). To this end, the stimuli used in our previous study [6] were also used in the current experiment and this also allowed a direct comparison to be made across the findings of both these studies. As the new research was also conducted in the UAE, we also took the opportunity to investigate the effects of two versions of the hijab that are common in this region. In most previous research, the hijab used was worn to cover all (or nearly all) of the entire head of each female image except for the face (e.g., [3, 12, 13]). But in the UAE, the hijab is worn in two ways, either to fully cover the head except for the face (*fully covered*, Fig 1A), or slightly away from the face (*partially covered*, Fig 1B), where each head is fully covered except for the face and areas around the forehead and each side of the face and head. Including both versions in our experiment enabled a more comprehensive assessment of the effects of the hijab on female facial attractiveness to Muslim men.

Under these conditions, the effects of the hijab were more likely to reflect accurately male perceptions of female facial attractiveness without being distorted by influences of Islamophobia. Indeed, because we were studying the perceptions of Muslim men in their native Muslim country (the UAE), if preference for one's own cultural group (cultural endogamy) exerted a major positive influence on perception of facial attractiveness in hijab-wearing women, influences of piety and devoutness to Islam within the UAE may lead participants to rate the facial attractiveness of hijab-wearing women more highly. However, if perception of female facial attractiveness by native Muslim men is not dominated by endogamy, and the status of our participants within their native country enabled them to feel assured in making assessments of female facial attractiveness based on perceptual, rather than cultural, factors, the findings of this experiment should provide a clear indication of the effect of the hijab on perceived facial attractiveness. In particular, if the hijab really is an effective limiter of female facial attractiveness perceived by native Muslim men, participants should perceive faces as substantially less attractive in images where the hijab is worn compared to when no hijab is present. From our previous findings with female participants [6], both types of hijab display (fully covered, partially covered) may produce this effect. In contrast, if the hijab is not an effective limiter of female facial attractiveness perceived by native Muslim men, wearing the hijab in either form should have no effect on participants’ perceptions of facial attractiveness compared to when no hijab is present. Indeed, in this situation, all types of display (fully covered, partially covered, and uncovered; see Fig 1) should produce similar levels of facial attractiveness. However, if differences in the way in which the hijab is worn (fully covered, partially covered) exert their own effects on facial attractiveness, this difference should be apparent when comparing perceptions between these two conditions.

## Method

### Participants

Following the considerable administrative processes required for recruiting male participants to view images of females in the UAE, sixty males, aged 18–25 years (*M* = 19.7, *SD* = 1.5), were selected to participate in the experiment. All participants were unpaid volunteers and were recruited via flyers posted in and around Zayed University in the UAE which provided a suitable site for the research. This was also the same population from which female participants had been recruited in the study by Sheen et al. [6]. The experiment was conducted from September to December, 2018, and all participants were practicing Muslims and reported being highly religious and native to and living in the U.A.E. These details were verified using official documentation and interviews to ensure that all participants satisfied the requirements of this study. All interactions with participants were conducted in Arabic and English. In addition, the religiosity of each participant was assessed after each experimental session using the Duke University Religion Index (DUREL; [30] which measured intrinsic religiosity, personal religious commitment, and religious motivation. Previous studies (e.g., [31–33]) have shown that the DUREL has a high test-retest reliability (intra-class correlation coefficient of 0.91), a high internal consistency (Cronbach’s α 0.78–0.91), and convergent validity with other established measures of religiosity (*r*’s = 0.71–0.86). Participants’ responses were based on a 1 (*strongly disagree*) to 5 (*strongly agree*) response range. Religiosity scores were high (*M* = 4.87; *SD* = 0.35, range = 3.67–5, mode = 5), indicating that participants rated themselves as highly religious.

All participants showed normal or corrected-to-normal visual ability, as determined by Bailey-Lovie [34] assessments (see [35]). A priori estimates of the required sample size were obtained using G*Power [36] for a statistical power of 0.95 at an α level of 0.05 and an effect size of .23. The power analysis indicated that a sufficient sample would be 51 participants, indicating that our sample of 60 was appropriately powered. All remaining aspects of the methodology and procedures of this experiment were designed to be the same as those used by Sheen et al. [6].

#### Ethics statement

This research was conducted in accordance with the recommendations of the Research Ethics Committee at Zayed University, with written informed consent from all participants, in accordance with the Declaration of Helsinki. The protocol was approved by the Research Ethics Committee at Zayed University.

### Stimuli

The stimuli used were identical to those used by Sheen et al. [6]. Stimuli were created using photographs of 20 Muslim women of Middle Eastern appearance to produce frontal views of the head of each woman against a constant neutral background. Each woman was aged between 21 and 40 years, reported wearing the hijab regularly in everyday life, and was photographed uncovered and wearing her own hijab in each of the two styles used in the experiment (Fig 1). These two styles are common in the UAE and were used to reflect the typical variety that exists in how the hijab is worn. Each woman was then presented in each of the three display conditions used in the experiment (Fig 1). In the *fully covered* condition (A), each head was covered completely with the exception of the face. In the *partially covered* condition (B), each head was also covered completely with the exception of the face but now an area was also visible around the forehead and on each side of the face and head. In the *uncovered* condition (C), no hijab was worn so that each head was completely uncovered. For each woman, it was important to use exactly the same facial image in each of the 3 display conditions. Therefore, for each woman, the uncovered image was used as the basis for the partially covered and fully covered images so that the hijab in each case could be superimposed digitally on the same facial image. In this way, we were able to produce 3 display conditions for each face where each face was presented unchanged apart from the hijab manipulations. All stimuli were shown at their natural full-size and in full colour, at a viewing distance of 60 cm. When asked at the end of the experiment, all participants reported that all images looked natural and that they were unaware that facial images were occasionally identical.

### Apparatus and design

All 60 stimuli were shown individually on a high-definition visual display linked to an Apple Macintosh computer. Stimuli were shown in a different random order for each participant, and presentations and responses were made via an SR response box interfaced with the computer which provided millisecond response-timing accuracy, controlled by Experiment Builder software (SR Research Ltd., Kanata, Ontario, Canada). Participants responded on each trial by pressing one of 7 keys interfaced with the computer, corresponding to a 7-point scale indicating how attractive they regarded each face (1 = *very unattractive* to 7 = *very attractive*). The rating of each response was the dependent variable of the experiment but, for completeness. response times were also recorded.

### Procedure

Participants took part individually in a sound-attenuated room and sat in front of the visual display. At the start of the session, each participant was informed that images of female faces would be displayed one at a time, together with a 7-point scale which they should use to rate carefully how attractive they regarded each face. Each image remained on the screen until a response was made, after which the next image appeared. Each session began with six practice stimuli to familiarize participants with the procedure before the experimental stimuli were shown.

## Results

**Fig 2:**
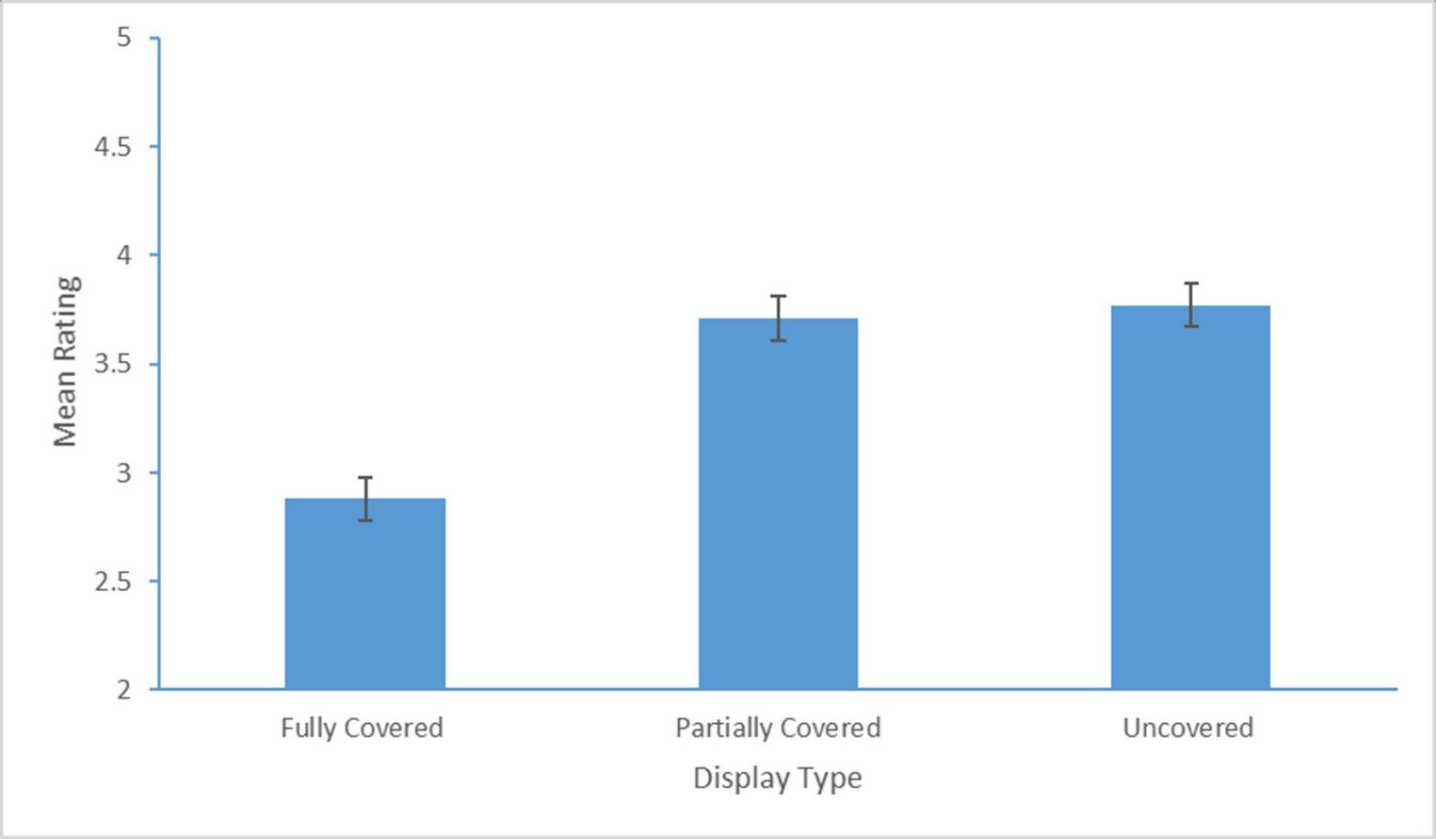
Mean facial attractiveness ratings (with standard error bars) for fully covered, partially covered, and uncovered images. The y-axis scale has been adjusted for clarity (ratings in the experiment were on a scale of 1–7).

Mean attractiveness ratings for each display condition are shown in Fig 2. A repeated measures ANOVA conducted on attractiveness ratings with the factor display condition (fully covered, partially covered, uncovered) using a Greenhouse-Geisser correction showed a significant main effect, *F*(1.60, 94.61) = 109.66, *p* < .0001, η^2^ = .65. Post-hoc comparisons using Bonferroni-corrected *t*-tests revealed that faces where heads were uncovered (*M* = 3.77, *SD* = .63) or partially covered (*M* = 3.71, *SD* = .59) were rated as equally attractive (differing by just 0.06, less than 1% of the available rating scale; *p*>.70) but both were rated as significantly more attractive than faces where heads were fully covered (*M* = 2.88, *SD* = .73; *p*s < .001). As a precaution, times taken to respond were also analyzed and showed similar response times for each of the 3 display conditions (Table 1), *F*(2, 118) = 1.90, *p* > .15, η^2^ = .03. This suggests that participants were similarly engaged in making responses to all 3 types of display.

**Table 1:** Mean reaction time (in milliseconds) for fully covered, partially covered, and uncovered images.

	Fully Covered	Partially Covered	Uncovered
Mean	3039	3235	3263
SD	1436	1468	1394

In line with the aim of our experiment, each participant showed a high level of religious belief and all participants rated their religiosity very highly (see *Participants* section). So it seemed unlikely that there would be enough variation to show a meaningful relationship between different levels of self-reported religiosity and attractiveness ratings. Nevertheless, we investigated the relationship between religiosity and attractiveness ratings in each of the three display conditions used in our study. A Shapiro-Wilk test of distribution normality for these religiosity ratings was significant, *S-W* = .41, *df* = 60, *p* < .0001, reflecting a skew towards high religiosity scores. Consequently, a Spearman correlation coefficient was used to evaluate the association between DUREL test scores and attractiveness ratings individually for each of the three conditions. No significant correlation was found in any condition (for all display conditions, *r* < .09, *p*>.50).

It was apparent that the pattern of performance observed for covered and partially covered images in the current study with male participants differed from that observed previously with female participants by Sheen et al. [6], and this was supported by a subsidiary analysis which compared the data across these two studies. A mixed design ANOVA, with factors participant gender (male, female) and display condition (fully covered, partially covered, uncovered), showed no main effect of participant gender, *F*(1,118) = .02, *p* = 0.90, $\eta_{p}^{2}$ = .00, but (using a Greenhouse-Geisser correction) showed a main effect of display condition, *F*(1.64, 193.82) = 88.03, *p* < 0.001, $\eta_{p}^{2}$ = .43, and, crucially, an interaction between participant gender and display condition, *F*(1.64, 193.82) = 31.89, *p* < 0.001, $\eta_{p}^{2}$ = .21.

Pairwise comparisons (Bonferroni-corrected *t*-tests) showed that uncovered images were rated equally across genders (for males, *M* = 3.77, *SD* = 0.63; for females, *M* = 3.75, *SD* = 1.00, *p*>.90, *d* = .02) and confirmed the different pattern across display conditions already reported in each study (the present study and the study by Sheen et al. [6]). Specifically, for male participants (the present study), faces where heads were uncovered and partially covered were rated as equally attractive (*M* = 3.77, *SD* = 0.63 vs *M* = 3.71, *SD* = 0.59, *p*>.40, *d* = .10) and both as more attractive than faces where heads were fully covered (*M* = 2.88, *SD* = 0.73, *p*s < .001, *d*s>1.2). For female participants (Sheen et al.), faces where heads were fully covered and partially covered were rated as equally attractive (*M* = 3.28, *SD* = 1.01 vs *M* = 3.28, *SD* = 1.01, *p*>.90, *d* = 0) and both as less attractive than faces where heads were uncovered (*M* = 3.75, *SD* = 1.00, *p*s < .001, *d*s = .47).

## Discussion

The purpose of this research was to investigate the effects of wearing the hijab on Muslim men’s perceptions of female facial attractiveness. This was achieved using images of women’s heads shown in three display conditions: fully covered (wearing the hijab so that each head was covered completely except for the face); partially covered (wearing the hijab so that each head was covered completely except for the face and areas around the forehead and on each side of the face and head); and uncovered (wearing no hijab so that each head was completely uncovered). Of particular importance is that the participants providing these perceptions were all practicing Muslim men living in their native Muslim country (the UAE) where Islam and the hijab are normal and universally accepted aspects of everyday life. As a result, perceptions by Muslim men of facial attractiveness in women wearing the hijab were unlikely to be affected by anti-Islamic feelings, thus avoiding negative influences that may have affected previous research on male perceptions of female facial attractiveness carried out in a non-Muslim country (the UK; [12, 13]). But despite this Islamic cultural environment and the high levels of religiosity of our participants, practicing Muslim men in this indigenous Muslim culture rated female faces when heads were uncovered or partially covered as equally attractive, and both as substantially more attractive than the same female faces when heads were fully covered by the hijab.

Considering the cultural nature of this study, it seems unlikely that, compared to uncovered and partially covered images, the lower facial attractiveness observed when heads were fully covered was produced by negative perceptions of religious affiliation. So why were covered images rated so lowly, and uncovered and partially covered images both rated so highly, especially given the considerable cultural differences that exist between these latter two types of facial display? One factor may be that Muslim men living in their native Muslim country are very aware of the intention of females in Muslim society to wear the hijab in order to restrict their physical attractiveness to men and, in the UAE, women who are fully covered by the hijab are often regarded as showing the greatest depth of faith (e.g., [1, 18]). This could help explain why fully covered images in our study produced the lowest attractiveness ratings of all, and the higher ratings observed for partially covered images, as both may reflect influences of a form of self-fulfilling prophecy [37] or confirmation bias [38] by the Muslim participants who took part. But even wearing the hijab away from the face can signify some degree of piety and religious conviction in Muslim society and a wish to reduce attractiveness to men, and this sign of intent is patently absent when women wear no covering at all. And yet our findings showed that partially covered and uncovered images produced practically identical ratings of facial attractiveness. It may be that facial attractiveness in uncovered images was also moderated, but this time by a perceived lack of religious commitment, and this served to lower the ratings given for this condition. But while further research may unravel the contributions of these and other influences, it is already apparent that such influences as perceived religious conviction and perceived lack of religious commitment, respectively, would be expected to suppress the facial attractiveness of female faces shown partially covered and with no covering at all, and so the higher attractiveness actually observed for both these conditions implicates a process that was sufficiently powerful to overcome both these potential effects of cognitive bias. Accordingly, if cognitive bias based on the perceived intentions of women wearing a hijab, or based on the perceived lack of religious commitment by uncovered women, affected participants’ ratings, this effect appears to have been relatively weak and other factors are likely to play an influential role in the effect of the hijab on facial attractiveness.

One such factor that deserves close consideration, especially when determining why partially covered and uncovered images produced the highest ratings of facial attractiveness, concerns the effects that the hijab may have on normal processes of facial perception (see also Sheen et al. [6]). In particular, a good deal of evidence suggests that humans process faces as integrated perceptual wholes rather than as collections of individual features [39, 40], and features external to the face (such as hair and ears) may play a crucial role in this process ([10, 41; see also 42]). Indeed, from the findings of Toseeb et al. [10, 41], wearing the hijab may produce substantial differences in the way faces are recognized, and external features may play an important role in facial processing which changes when these features are not visible. In light of this evidence, it seems likely that external features may also affect perception of facial attractiveness, and some support for this view does exist. Kramer and Ward [43], for example, found that images of women’s faces displayed in full produced greater discrimination of facial characteristics associated with facial attractiveness (e.g., physical health) than when the same faces were shown with only their internal features displayed (e.g., eyes, nose, mouth). Consequently, as the hijab evidently has the ability to reduce substantially the visibility of external features of faces, it seems plausible that wearing the hijab may affect facial attractiveness by disrupting normal processes of human facial perception.

The occlusion of external features offers a perceptual contribution to explaining the low level of facial attractiveness observed for fully covered images in our study and, relatedly, the high level of facial attractiveness observed for uncovered images. But although partially covered images also involved wearing the hijab and the occlusion of external features, participants rated facial attractiveness higher for partially covered images than for fully covered images, and practically identical to that observed for uncovered images. From a perceptual perspective, this increase in attractiveness for partially covered images compared to fully covered images may be explained by the increased featural information provided. In particular, while fully covered images provided an effective occlusion of all parts of the head outside the circumference of the face and jawline, partially covered images revealed considerable amounts of the head above and around the forehead, around the temples, and at other lateral locations, and allowed a much greater amount of hair to be seen. Indeed, for fully covered images, complete occlusion of hair may provide an especially influential contribution to the reduction in attractiveness observed relative to uncovered images, and the absence of this effect when images were only partially occluded. Hair is one of the most controllable aspects of physical attractiveness and many women (and men) spend a great deal of money, time, and effort grooming their hair in order to appear more attractive to others [26]. Arguments for the role of hair in attractiveness have been presented in several studies, and hair provides a normally visible indication of a woman’s youth and health, which can increase attractiveness substantially [26, 44, 45]. Indeed, in the present study, partially covered and uncovered images revealed similar amounts of hair around the face, and this may explain why both types of image produced the same higher ratings of facial attractiveness relative to the fully covered condition. But, again, it should be noted that partially covered and uncovered images produced essentially identical ratings of facial attractiveness despite the fact that, for native residents in the UAE, women wearing the hijab are regarded as showing greater depth of faith than women who are uncovered. The effect of the hijab on the *perceptual* processing of women’s faces, therefore, may produce a profound and powerful influence on facial attractiveness.

The purpose of this study was to investigate the effects of the hijab on the facial attractiveness of women perceived by practising Muslim men in their native Muslim country, and the effects we observed provide new insight into the use of religious veiling for regulating and restricting female sexuality. In particular, as Pazhoohi et al. [9] suggest (see also [46]), the personal and social aspects of religious veiling have been studied extensively but the actual function of religious veiling is still not clear. From an evolutionary perspective, reducing female attractiveness may act as a mate guarding strategy [7, 26] which safeguards a man’s access to a mate while simultaneously preventing advances from male rivals. Indeed, although mate guarding can serve a number of different purposes (for a review, see [8]), its primary goal is likely to be to control female sexuality and thus reduce incidences of infidelity and cuckoldry [7]. In a similar vein, Pazhoohi et al. [9] argue that if religious veiling is used primarily to guard female mates from male rivals, an adaptive consequence of this may be a greater use of veiling in environments where paternal investment is higher, and so where more effort should be made to guard female mates. Pazhoohi et al.’s findings [9] support this argument. But the findings of the present study suggest that when men in an Islamic country enforce the wearing of the hijab, these men are also likely to be aware of the effect that this has on perceived facial attractiveness. Thus, when used as a mate-guarding strategy, the function of the hijab is likely to be more than a cultural signal of rejection to other men as it evidently affects fundamentally how female faces are actually perceived by men. Indeed, our findings suggest that men enforcing the wearing of the hijab are also likely to be aware of the greater effectiveness of hijabs that fully cover the head and hair (fully covered images in our study) and that slight shifts in the positioning of the hijab (partially covered images in our study) produce a less effective suppression of female facial attractiveness to male observers (and potential male rivals).

It is interesting to note that the pattern of effects observed for male participants in this study differs from that observed for female participants by Sheen et al. [6]. In particular, Sheen et al. found that native Muslim females in the UAE regarded female faces as no more attractive in partially covered images than in fully covered images, and faces in both these images were regarded as less attractive than in uncovered images. Thus, Muslim female perceptions of female facial attractiveness appear to be affected negatively by the presence of any hijab (facial attractiveness was equally lowest for covered and partially covered images) whereas the current study suggests that Muslim male perceptions of female facial attractiveness are affected positively by the availability of featural information even when a hijab is present (facial attractiveness was equally highest for uncovered and partially covered images). This difference between perceptions of female facial attractiveness by Muslim women and men may be explained by the effect that the hijab has on the way in which Muslim women and men perceive facial attractiveness. When considering the perceptions made by Muslim women, Muslim women in society are generally obliged culturally to wear the hijab to restrict facial attractiveness only in the presence of men outside their immediate family. So, when seeing images of women wearing any hijab, even a hijab that only partially covers the head, Muslim women may see a clear and explicit cultural intention by the wearer to reduce facial attractiveness, and this may inspire both types of hijab to produce a similar, less attractive image to Muslim female observers (e.g., [3, 47]). In contrast, for Muslim men, the importance of featural information may dominate perceptions of female facial attractiveness which overcomes cultural considerations concerning wearing the hijab. But this gender difference need not mean that Muslim women are unaware of the effect that wearing the hijab has on their own facial attractiveness to men. Indeed, by observing men’s reactions, Muslim women may become readily aware that how they wear the hijab [48], either fully or partially covered, allows them to have considerable control over their physical attractiveness to men even when the hijab is worn. In particular, wearing hijabs that partially cover the head can increase female facial attractiveness to men while also satisfying the conservative norms of their culture.

In sum, the goal of this study was to investigate the effectiveness of the hijab for limiting the facial attractiveness of women to practising Muslim men in their native Muslim country (the UAE). The findings suggest that wearing the hijab per se is not effective at this task, and that the facial attractiveness of women to men relative to when no hijab is worn is reduced only when the hijab covers all areas of the head outside the circumference of the face and jawline. We argue that this pattern of effects is not consistent with influences of anti-Islamic feeling or cultural endogamy and a major influence occurs because being fully covered by the hijab occludes external features, especially the hair and parts of the head and face, which normally contribute to perceiving human facial attractiveness. Thus, while wearing the hijab may sometimes be motivated by a cultural desire to suppress female attractiveness to men (e.g., [7–9]), these new findings suggest that not all hijab wearing serves this purpose, and that female facial attractiveness, even for practising Muslim men living in their native Muslim country, is not reduced simply by wearing this garment. From the findings we report, slight changes to the positioning of the hijab can produce perceptions of facial attractiveness that are no lower than when no hijab is worn, and this has interesting implications for when the hijab is worn to reduce the attractiveness of women to men.

Finally, two aspects of the present study should be emphasized. First, the current research contributes to understanding the effects of the hijab on facial attractiveness by investigating the perceptions of young Muslim men in their native Muslim country. But this research now needs to be extended to older age groups of Muslim men who may differ in their perceptions of female facial attractiveness. A good deal of evidence already suggests that perception of facial attractiveness is similar across different age groups ([49, 50; see also 51]) and different cultures [52–55] but it remains to be seen how the age of the beholder affects perceptions of facial attractiveness in hijab-wearing women. Second, changes in facial attractiveness produced by the hijab are likely to lead to other effects on the way in which women are regarded in society but these other effects were not part of the current study. In particular, it is largely accepted that a person’s physical appearance is the characteristic most obvious to others in social interactions (e.g., [56, 57]) and people who are considered physically attractive are more likely to be perceived as possessing a number of socially desirable traits, such as intelligence, competence, employability (e.g., [57–62]), greater socio-economic status (e.g., [19]), and even greater innocence in a court of law (see [63]). When considering the full effect of wearing the hijab on facial attractiveness, therefore, it seems likely that the way in which the hijab is worn will produce widespread influences on the social perceptions of the women who wear this garment. Accordingly, an important avenue for future research is to investigate the ways in which different levels of head covering (fully covered, partially covered, uncovered) affect social judgments more generally by looking at perceptions of the personal characteristics of females, such as intelligence, competence, and employability, and the extent to which these are associated with perceptions of facial attractiveness produced by the hijab. The effects of the hijab on the perception and treatment of women in society are of worldwide social importance and a body of research that allows a comprehensive understanding of these undoubtedly complex effects would be of considerable international social value.

## Supporting information

S1 Data Male attractiveness data. (XLSX)
